# Supplementary figures and images for: Müller cell glutamine metabolism links photoreceptor and endothelial injury in diabetic retinopathy
Source: Life Sci Alliance. 2025 Nov 20;9(2):e202503434. doi: 10.26508/lsa.202503434 (PMC12634822; doi:10.26508/lsa.202503434)

## SLC38A3

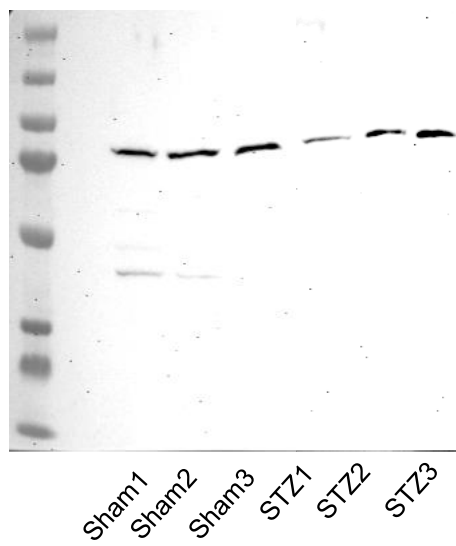

## BACT

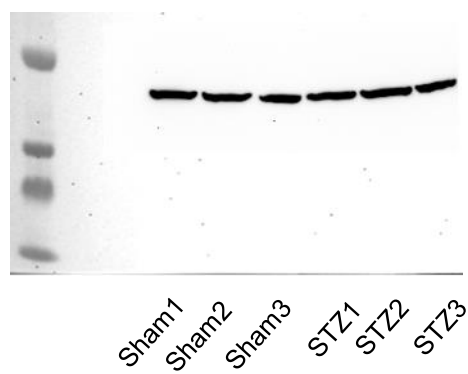

Supplement: Supplementary file 6 [file LSA-2025-03434_SdataF4.2.pdf]

## COL6

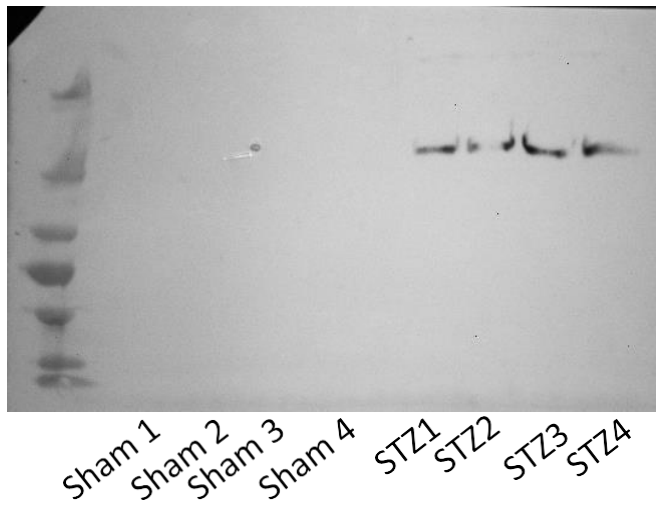

## BACT

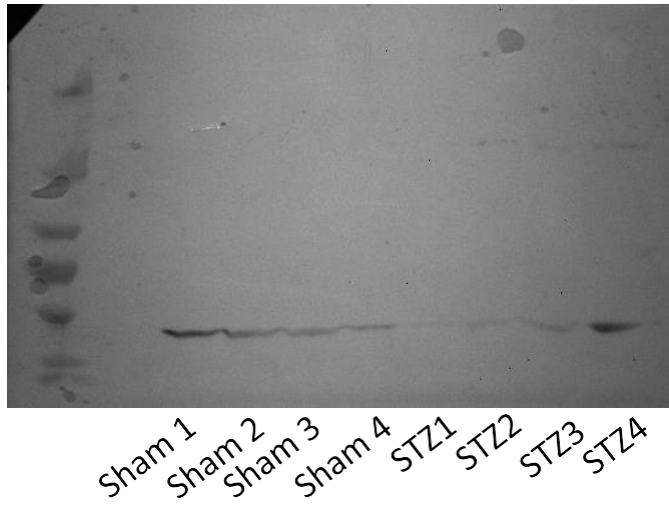

## BACT

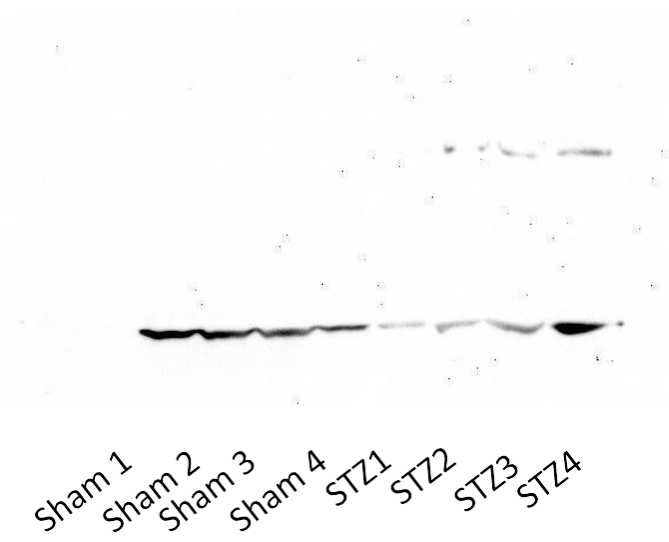

Supplement: Supplementary file 11 [file LSA-2025-03434_SdataF7.2.pdf]
