# Supplementary material for: Müller cell glutamine metabolism links photoreceptor and endothelial injury in diabetic retinopathy
Source: Life Sci Alliance. 2025 Nov 20;9(2):e202503434. doi: 10.26508/lsa.202503434 (PMC12634822; doi:10.26508/lsa.202503434)

GS

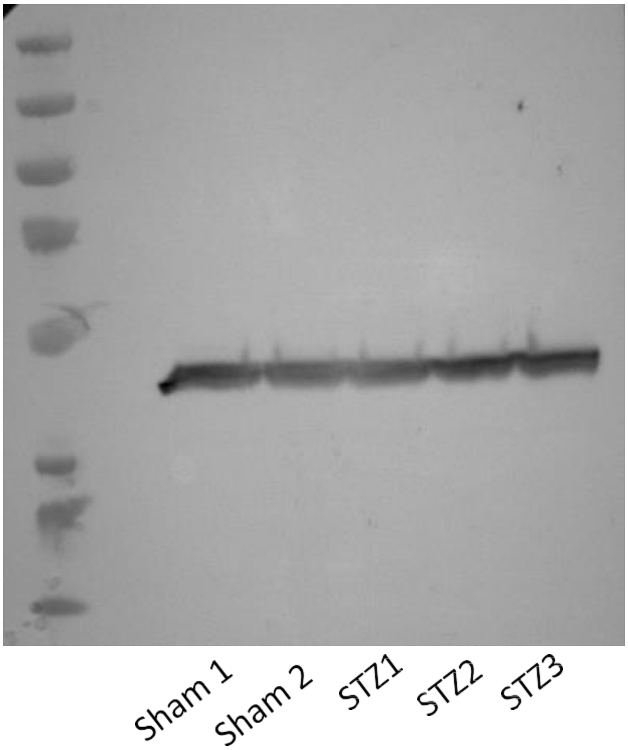

BACT

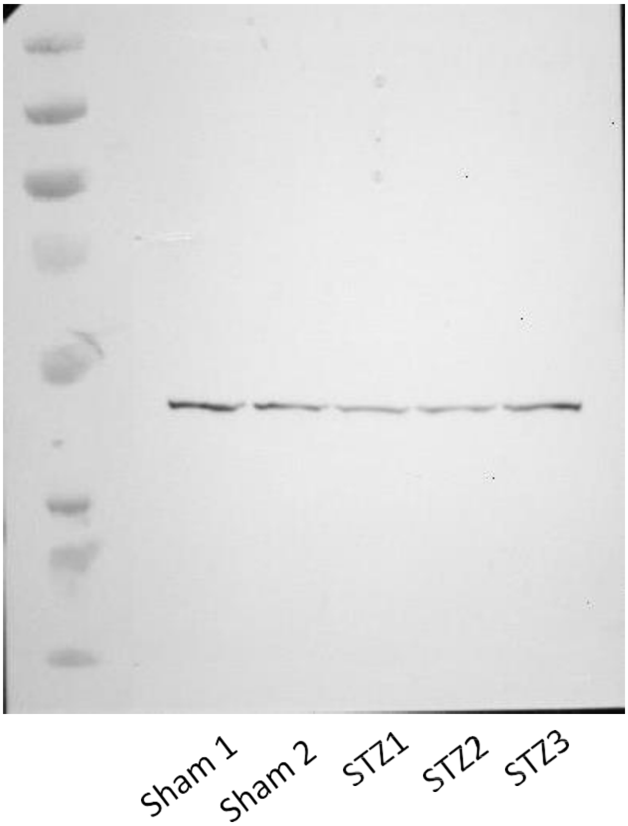

GS

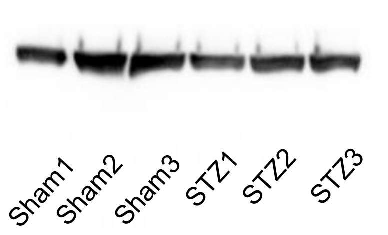
Sham1
Sham2
Sham3
STZ1
STZ2
STZ3

BACT

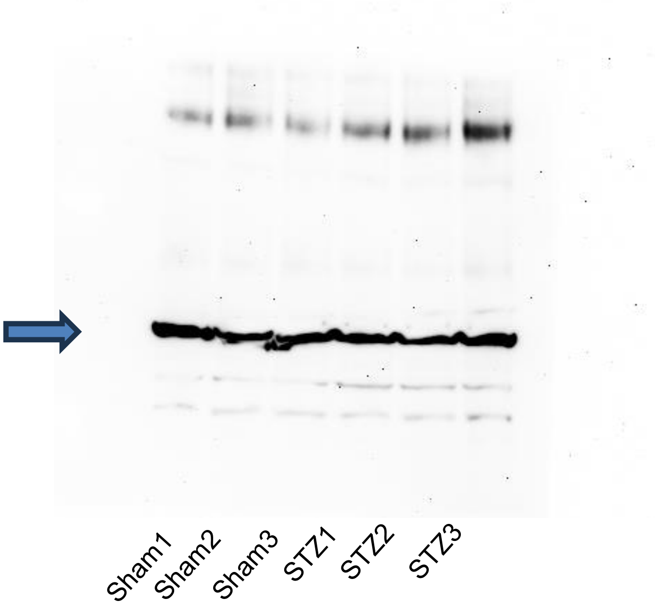
Sham1
Sham2
Sham3
STZ1
STZ2
STZ3

GLAST

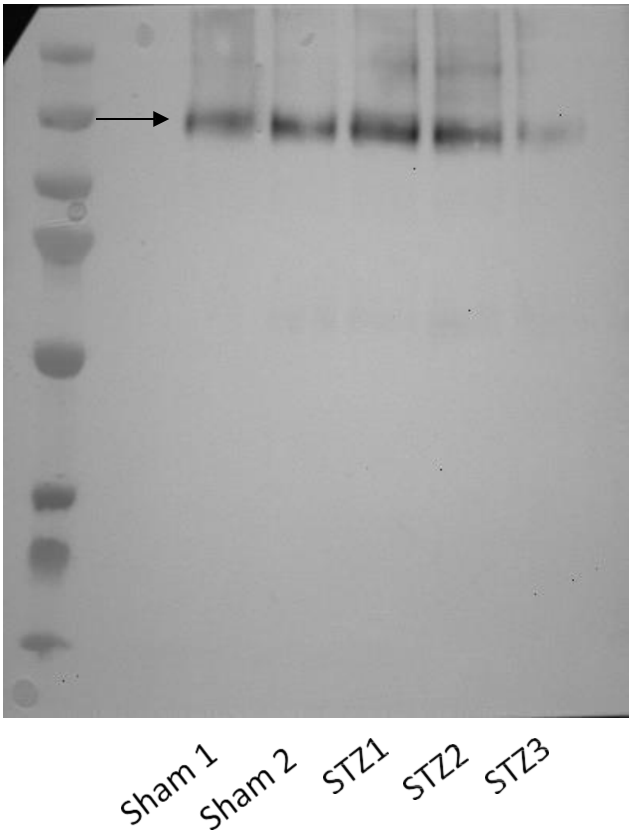

BACT

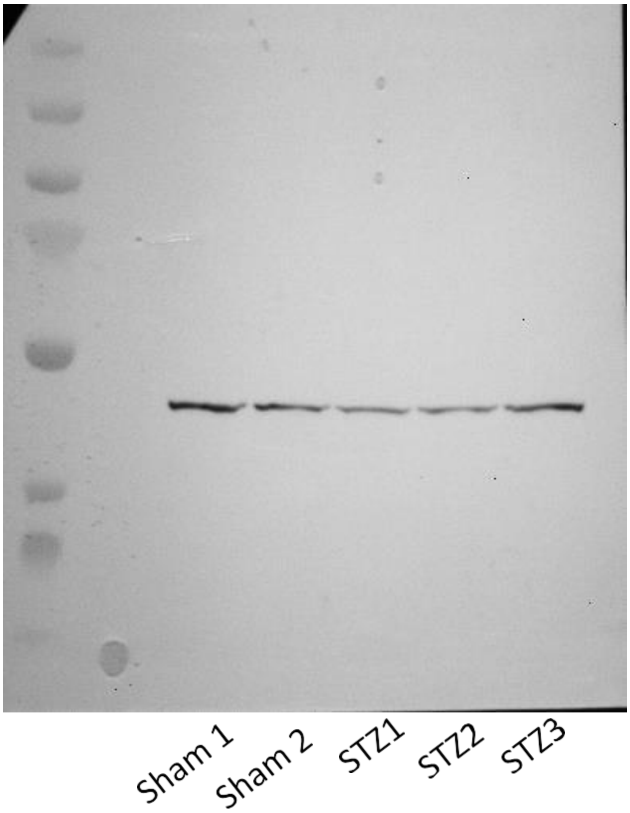

Supplement: Supplementary file 8 [file LSA-2025-03434_SdataF5.2.pdf]
